# Ontogeny and ecological significance of metabolic rates in sea turtle hatchlings

**DOI:** 10.1186/s12983-022-00451-2

**Published:** 2022-02-05

**Authors:** Christopher R. Gatto, T. Todd Jones, Brittany Imlach, Richard D. Reina

**Affiliations:** 1 School of Biological Sciences, Monash University, 25 Rainforest Walk, Clayton, VIC 3800 Australia; 2 NOAA Fisheries, Pacific Islands Fisheries Science Center, Honolulu, HI USA; 3 Centre for Comparative Medicine, The University of British Columbia, Vancouver, BC Canada

**Keywords:** Oxygen consumption, Metabolism, Sea turtle, Ontogeny, Life history, Aerobic scope

## Abstract

**Background:**

Sea turtle hatchlings must avoid numerous predators during dispersal from their nesting beaches to foraging grounds. Hatchlings minimise time spent in predator-dense neritic waters by swimming almost continuously for approximately the first 24 h post-emergence, termed the ‘frenzy’. Post-frenzy, hatchling activity gradually declines as they swim in less predator-dense pelagic waters. It is well documented that hatchlings exhibit elevated metabolic rates during the frenzy to power their almost continuous swimming, but studies on post-frenzy MRs are sparse.

**Results:**

We measured the frenzy and post-frenzy oxygen consumption of hatchlings of five species of sea turtle at different activity levels and ages to compare the ontogeny of mass-specific hatchling metabolic rates. Maximal metabolic rates were always higher than resting metabolic rates, but metabolic rates during routine swimming resembled resting metabolic rates in leatherback turtle hatchlings during the frenzy and post-frenzy, and in loggerhead hatchlings during the post-frenzy. Crawling metabolic rates did not differ among species, but green turtles had the highest metabolic rates during frenzy and post-frenzy swimming.

**Conclusions:**

Differences in metabolic rate reflect the varying dispersal stratagems of each species and have important implications for dispersal ability, yolk consumption and survival. Our results provide the foundations for links between the physiology and ecology of dispersal of sea turtles.

## Background

The majority of oviparous reptiles provide minimal parental care to their offspring [1]. Thus, offspring must emerge from the nest and disperse unassisted. Consequently, smaller and slower offspring may be at greater risk of predation than offspring that are larger and faster [2–4]. Sea turtle hatchlings have high mortality rates compared to other reptiles because of high predation rates during their prolonged dispersals [4, 5]. In particular, predation rates are highest where predator densities are highest, specifically when the hatchlings crawl from the nest to the ocean and when the hatchlings swim in near-shore waters [5]. To reduce the time spent in predator-dense zones, hatchlings undergo a period of hyperactivity for approximately the first 24 h post-emergence. During this 24 h period of hyperactivity termed the ‘frenzy’ [6], hatchlings swim almost continuously and exhibit high thrust production as they quickly disperse from the natal beach and surrounding waters [7–17].

While an effective strategy for predator evasion, the continuous swimming and high thrust production of the ‘frenzy’ is energetically demanding [7, 15, 18, 19]. During the frenzy, hatchling swimming activity can be broken into three phases: the rapid fatigue phase when oxygen consumption is initially high and quickly declines; followed by the slow fatigue phase when oxygen consumption rates continue to drop, but at a slower rate; and lastly the sustained effort phase when oxygen consumption is relatively stable [7]. As most hatchlings survive solely on residual yolk reserves during dispersal, maintaining high activity levels and high energy consumption rates may place hatchlings at greater risk of fatigue and resource depletion before reaching foraging grounds compared to hatchlings with lower energy demands [2, 18, 20]. Hatchling activity levels are highest during the initial dispersal across the beach and through neritic waters where predator-densities are highest [8, 21], and once hatchlings enter deeper, pelagic waters, the total time that they spend swimming per day gradually decreases [8, 21]. Sea turtle species differ in the rate at which they shift their swimming activity and behaviour [8, 9, 22], and these differences are often attributed to variation in life history among species. For example, flatback, *Natator depressus*, hatchlings remain completely within neritic waters during dispersal and they exhibit smaller reductions in swimming activity levels compared to other species [22], potentially in order to avoid or escape predators in these predator-dense waters [23]. Differences in swimming activity have also been observed among populations, providing further support that divergence in life history and selective pressures drive variation in swimming activity [24].

While the ontogeny of swimming activity and the change in swimming behaviour as hatchlings age between frenzy and post-frenzy swimming has been studied previously [7–13, 16, 17, 21–23], the ontogeny of metabolic rates remain relatively unstudied [18, 19]. This difference is likely because hatchling metabolic rates (MRs) are typically measured by estimating oxygen consumption, which requires specialised equipment. More common are proxies of metabolic rate that include direct measures of swimming behaviour, flipper stroke rates, and swimming bout durations [7, 18]. However, metabolic rates are key measures of the energetic capacity of hatchlings to disperse, determining how long they can remain active. Hatchlings that have higher metabolic rates may have a greater ability to swim quickly, but also may consume their yolk reserves more quickly than hatchlings with lower metabolic rates. Determining how sea turtle hatchlings utilise energy is critical for understanding limits of hatchling dispersal, foraging, and growth, which has important implications for population dynamics and ecology of dispersal.

While few studies have been conducted on this topic, these preliminary studies suggest that the ontogeny of sea turtle hatchling metabolic rates varies among species [18, 19]. To further our understanding, we measured and compared the metabolic rates of five sea turtle species during the frenzy and post-frenzy. We measured oxygen consumption during rest (resting metabolic rate, RMR) when frenzy and post-frenzy hatchlings were quiescent; crawling metabolic rate (CMR) when only frenzy hatchlings were actively and continuously crawling on sand; routine swimming (active metabolic rate, AMR) when frenzy and post-frenzy hatchlings were actively and continuously swimming of their own volition; and maximal metabolic rate (MMR) when frenzy and post-frenzy hatchlings were being stimulated to swim with maximum effort. Each measure reflects specific energy requirements to support the various ecological demands during the frenzy and post-frenzy phases: RMR reflects the energy requirements to support breathing and other basic physiological functions such circulating blood [25]; CMR represents the energy requirements to fuel hatchling dispersal from the nest to the ocean; AMR represents normal activity associated with foraging and general locomotion [26]; and MMR represents the maximum energy production capable by an individual turtle, such as when threatened by a perceived predator [18, 26]. We measured oxygen consumption to compare differences in metabolic rates among behavioural stages (frenzy, post-frenzy), activity levels (RMR, CMR, AMR, MMR) and species (olive ridley *Lepidochelys olivacea*, green *Chelonia mydas*, flatback *Natator depressus*, leatherback *Dermochelys coriacea*, and loggerhead *Caretta caretta* sea turtles) that determine each species’ energetic capacity to disperse from nesting beaches to foraging grounds. Additionally, we compared each species’ aerobic scope i.e., the ability of an individual to elevate its aerobic metabolic rate above resting. We hypothesised that metabolic rates and aerobic scopes vary among activity levels, behavioural stages and species in a manner that matches the species’ and population’s early life history stratagems. Specifically, we hypothesised that species with greater predation pressure during the frenzy would exhibit higher frenzy metabolic rates when crawling and swimming than species with lower predation pressures. We also hypothesised that post-frenzy, species with shorter dispersal migrations, such as flatbacks, would exhibit an earlier decrease in metabolic rates than species that undertake longer dispersal migrations. We aimed to then evaluate any differences in the context of the life history patterns and ecology.

## Results

### Overall variation in metabolic rates with activity level, behavioural stage and species

Hatchling mass and test temperatures for each species and location are reported in Tables 1 and 2, respectively. Hatchling MRs (Table 3) varied significantly with behavioural stage, activity and species (Table 4). Hatchling ID nested within location and species, and test temperature explained 76% and 24% respectively of the variation in metabolic rate. The interactions between activity and species, activity and behavioural stage, and among all three fixed effects were significant. Thus, we also evaluated differences among and within species, activity and behavioural stage separately. We report the results of mass-specific metabolic rate comparisons below.

**Table 1:** Hatchling mass and the number of hatchlings tested (mean ± SD, N) for each species, location, age and respirometry technique in this study

Species	Olive ridley (*Lepidochelys olivacea*)	Flatback (*Natator depressus*)	Leatherback (*Dermochelys coriacea*)	Loggerhead (*Caretta caretta*)	Green (*Chelonia mydas*)
Population/s	Australia	Australia	USA	USA	^a^USA ^b^Malaysia
Frenzy	Closed: 16.5 ± 0.2 g, 74	Closed: 40.4 ± 0.3 g, 80	Open: 44.9 ± 0.7 g, 27	Open: 18.4 ± 0.4 g, 21	Closed: 24.6 ± 0.2 g, 6^a^ Open: 24.7 ± 0.4 g, 24^a^ Open: 21.4 ± 0.2 g, 95^b^
6 d				Closed: 16.8 ± 0.2 g, 5	
7 d			Open: 45.2 ± 1.11 g, 10	Open: 20.9 ± 0.7 g, 10	Open: 26.8 ± 0.6 g, 8^a^
12 d				Open: 20.4 ± 0.9 g, 15	
20 d			Closed: 68.0 ± 5.5 g, 4		
22 d					Open: 28.9 g, 1^a^
23 d			Closed: 61.6 ± 3.3 g, 6		Open: 31.2 ± 0.6 g, 6^a^
25 d					Open: 37.3 ± 1.5 g, 7^a^
26 d					Open: 35.3 g, 1^a^
28 d	Closed: 19.4 ± 0.3 g, 70	Closed: 63.3 ± 0.5 g, 79			
31 d				Open: 35.4 ± 2.5 g, 3	
43 d				Closed: 60.7 ± 7.95 g, 2	
44 d			Closed: 99.2 g, 1		
45 d			Open: 70.2 ± 1.96 g, 19		
50 d			Open: 94.0 g, 1		
51 d				Closed: 89.9 g, 2	
52 d				Closed: 53.7 g, 1	

Only green turtles were tested from two different locations, denoted with superscript letters

**Table 2:** The air temperature (RMR & CMR) or water temperature (AMR & MMR) that hatchling oxygen consumption was measured at for each species, location and activity level

	Olive ridley	Flatback	Leatherback	Loggerhead	Green
	Australia	Australia	USA	USA	^a^USA ^b^Malaysia
RMR	Closed: 25 °C	Closed: 25 °C	Open: 23.5 ± 0.6 °C	Open: 24 ± 0.8 °C	Closed: 27.5 ± 1.2°C^b^ Open: 23.9 ± 0.5°C^a^
CMR			Open: 23.5 ± 0.6 °C	Open: 24 ± 0.8 °C	Open: 23.9 ± 0.5°C^a^
AMR			Closed: 22.5 ± 0.2 °C Open: 22.5 ± 2.7 °C	Closed: 28.8 ± 0.6 °C Open: 23.6 ± 1.5 °C	Closed: 28.4 ± 0.9°C^a^ Open: 24.6 ± 0.7°C^a^
MMR	Closed: 26.3 ± 0.4 °C	Closed: 26.3 ± 0.4 °C			Closed: 26.6 ± 1°C^b^

Only green turtles were tested from two different locations, denoted with superscript letters

**Table 3:** Olive ridley, flatback, leatherback, loggerhead and green sea turtle hatchlings resting metabolic rate (RMR), crawling metabolic rate (CMR), metabolic rate during routine swimming (AMR) and maximal metabolic rate (MMR) during the frenzy and post-frenzy

		Whole animal					Mass-specific				
		Olive ridley (μL O_2_ min^−1^)	Flatback (μL O_2_ min^−1^)	Green (μL O_2_ min^−1^)	Leatherback (μL O_2_ min^−1^)	Loggerhead (μL O_2_ min^−1^)	Olive ridley (μL O_2_ g^−0.67^ min^−1^)	Flatback (μL O_2_ g^−0.67^ min^−1^)	Green (μL O_2_ g^−0.67^ min^−1^)	Leatherback (μL O_2_ g^−0.67^ min^−1^)	Loggerhead (μL O_2_ g^−0.67^ min^−1^)
Frenzy	RMR	30 ± 2.06, n = 74	122.54 ± 4.5, 80	79.2 ± 3.4, 103	313.55 ± 35.93, 8	63.73 ± 6.45, 3	4.59 ± 0.31	10.3 ± 0.38	10.17 ± 0.45	23.76 ± 2.46	9.45 ± 1.06
	CMR			228.1 ± 95.85, 8	377.09 ± 47.14, 6	201.47 ± 32.28, 7			26.62 ± 11.09	28.82 ± 3.33	28.19 ± 4.6
	AMR			445.17 ± 26.43, 14	385.92 ± 36.87, 13	253.19 ± 15.2, 11			52.57 ± 3.34	30.84 ± 2.86	36.33 ± 2.24
	MMR	121.3 ± 6.88, 71	280.93 ± 18.83, 79	518.44 ± 14.46, 90			18.42 ± 0.99	23.6 ± 1.56	66.68 ± 1.9		
	Mass (g)	16.46 ± 0.44	40.39 ± 0.43	22.17 ± 0.52	44.91 ± 0.52	18.39 ± 0.4					
Post-frenzy	RMR	26.89 ± 1.55, 70	75.01 ± 1.82, 79	156.09 ± 23.06, 11	238.3 ± 28.95, 6	131.32 ± 53.94, 15	3.7 ± 0.22	4.67 ± 0.12	13.09 ± 1.94	13.94 ± 1.42	18.91 ± 8.61
	AMR			392.89 ± 68.98, 23	235.21 ± 12.84, 32	197.79 ± 29.82, 24			37.24 ± 6.69	15.18 ± 0.97	19.65 ± 2.26
	MMR	78.98 ± 4.53, 70	373.35 ± 18.52, 79				10.83 ± 0.62	23.02 ± 1.08			
	Mass (g)	19.39 ± 0.53	63.32 ± 0.58	37.04 ± 1.95	63.45 ± 1.89	29.33 ± 3.81					

Values are given as μL O_2_ min^−1^ (whole animal) and μL O_2_ g^−0.67^ min^−1^ (mass-specific) ± standard error

**Table 4:** Results from linear mixed effects model evaluating the effect of activity, behavioural stage, species and their interactions on oxygen consumption

	F-value	Df	*p*-value
Activity	**265.16**	**3**	** < 0.001**
Behavioural stage	**36.43**	**4**	** < 0.001**
Species	**166.93**	**4**	** < 0.001**
Activity: behavioural stage	**10.97**	**4**	**< 0.001**
Activity: species	**22.43**	**6**	**< 0.001**
Behavioural stage: species	**7.55**	**6**	** < 0.001**
Activity: behavioural stage: species	**10.86**	**1**	**< 0.001**

Significant relationships are highlighted in bold

### Change in oxygen consumption between behavioural stages

**Fig. 1:**
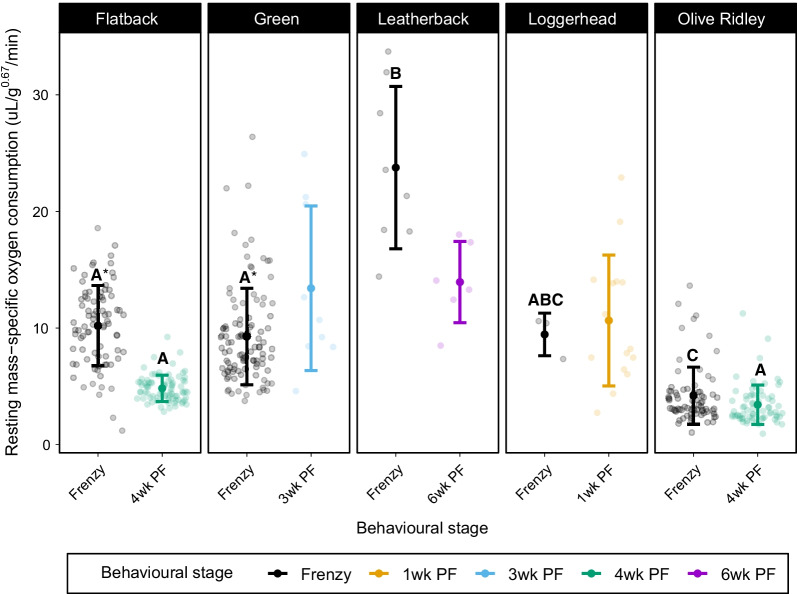
Mean resting mass-specific metabolic rate (μL O_2_ min^−1^ g^−0.67^) of sea turtle hatchlings during the frenzy and post-frenzy. Error bars represent 95% confidence intervals. Statistical differences between frenzy and post-frenzy resting metabolic rates within species are signified with *. Letters represent differences among species’ resting metabolic rates during the frenzy and post-frenzy, separately. Statistical differences were determined using mixed-effects models and Tukey’s pairwise comparisons

Within the activity analyses, RMR (i.e., when hatchlings were quiescent) did not differ between the frenzy and 1-week post-frenzy in loggerheads (z =  − 0.09, *p* = 1), 4-weeks post-frenzy in olive ridleys (z =  − 1.43, *p* = 0.61), or 6-weeks post-frenzy in leatherback hatchlings (z =  − 2.12, *p* = 0.21). However, flatbacks (z =  − 7.11, *p* < 0.0001) had higher RMR during the frenzy compared to 4-weeks post-frenzy, as did green hatchlings during the frenzy compared to 3-weeks post-frenzy (z = 2.93, *p* = 0.03; Fig. 1).

**Fig. 2:**
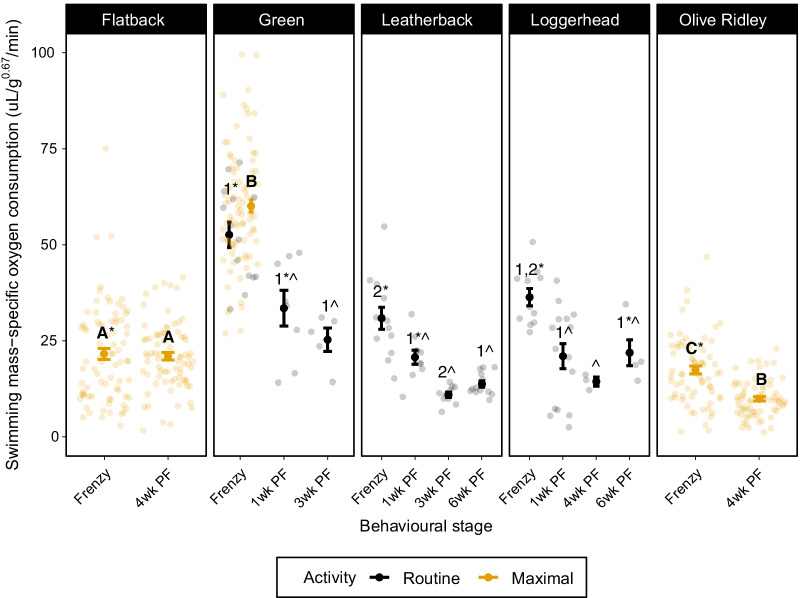
Mean mass-specific metabolic rate (μL O_2_ min^−1^ g^−0.67^) of swimming sea turtle hatchlings during the frenzy and post-frenzy. Error bars represent 95% confidence intervals. We present measurements made during routine swimming (circles with solid error bars) and maximal swimming (triangles with dashed error bars). Statistical differences between frenzy and post-frenzy metabolic rates within species are signified with the symbols * and ^. Numbers represent statistical similarities among species’ routine swimming metabolic rates within each behavioural stage, respectively. Letters represent statistical similarities among species’ maximal metabolic rates during the frenzy and post-frenzy, respectively. Statistical differences were determined using mixed-effects models and Tukey’s pairwise comparisons. Two outliers (Green frenzy: 126.97 μL O_2_ min^−1^ g^−0.67^, Green 3wk post-frenzy: 159.87 μL O_2_ min^−1^ g^−0.67^) were omitted from the figure to facilitate easier comparisons among behavioural stages and species. Both outliers were included in the analysis

During routine swimming, when hatchlings were allowed to swim continuously of their own volition, frenzied leatherback hatchling AMR did not differ from the AMR of hatchlings 1-week post-frenzy (z = -1.78, *p* = 0.38), but was higher than the AMR of hatchlings both 3-weeks post-frenzy (z =  − 3.85, *p* = 0.001) and 6-weeks post-frenzy (z =  − 4.01, *p* < 0.001; Fig. 2). Post-frenzy leatherback AMR did not differ among age groups: 1- and 3-weeks (z = 2.49, *p* = 0.09), 1- and 6-weeks (z = 2.24, z = 0.16), and 3- and 6-weeks post-frenzy (z =  − 0.78, *p* = 0.94). Frenzied green hatchling AMR did not differ from AMR 1-week post-frenzy (z = 2.18, *p* = 0.19), but was higher than AMR 3-weeks post-frenzy (z =  − 2.97, *p* = 0.03). Green hatchling AMR did not differ between 1- and 3-weeks post-frenzy (z = 0.87, *p* = 0.91). In loggerhead hatchlings, frenzy AMR was higher than 1-week (z =  − 3.3, *p* = 0.008) and 4-weeks post-frenzy (z =  − 2.76, *p* = 0.046), but did not differ from AMR 6-weeks post-frenzy (z =  − 1.85, *p* = 0.34). Post-frenzy AMR did not differ between 1- and 4-weeks post-frenzy (z = 0.77, *p* = 0.94), 1- and 6-weeks post-frenzy (z =  − 0.69, *p* = 0.96) or 4- and 6-weeks post-frenzy (z =  − 1.15, *p* = 0.78; Fig. 2).

During maximal swimming, both olive ridleys (z =  − 10.26, *p* < 0.0001) and flatbacks (z =  − 3.64, *p* = 0.003) had higher MMR during the frenzy compared to 4 weeks post-frenzy (Fig. 2).

### The effect of activity level on oxygen consumption by species

**Fig. 3:**
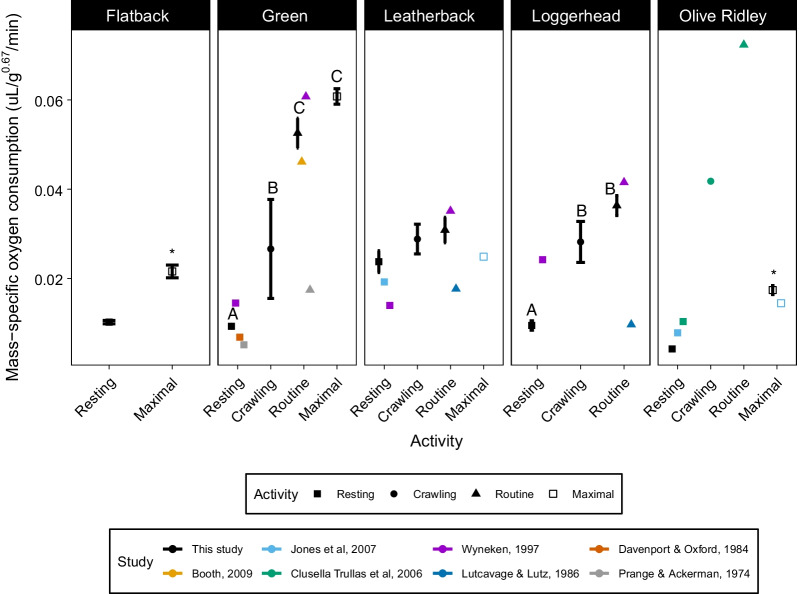
Comparison of mean sea turtle hatchling metabolic rates (μL O_2_ min^−1^ g^−0.67^) at different activity levels during the frenzy. Error bars represent 95% confidence intervals and we also report data from earlier studies on hatchling metabolic rates. We denote statistical differences between two activity levels within species with * and among 3 or more activity levels with letters. We converted measurements in previous studies from a mass exponent of 1 to an exponent of 0.67 to correct for allometric relationships between metabolic rate and hatchling mass [79]. Statistical differences were determined using mixed-effects models and Tukey’s pairwise comparisons. Data from Booth [7] was collected over the first 24 h of the frenzy but we only included data from the first 2 h of the frenzy

**Fig. 4:**
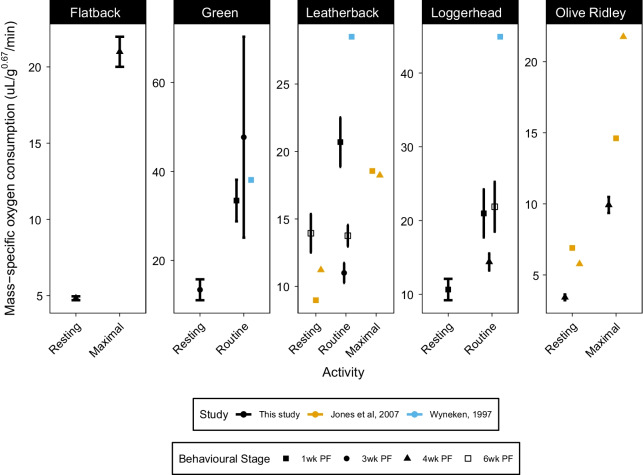
Comparison of mean sea turtle hatchling metabolic rates (μL O_2_ min^−1^ g^−0.67^) at different activity levels post-frenzy. Error bars represent 95% confidence intervals and we also report data from earlier studies on hatchling metabolic rates. We denote statistical differences among activity levels within species with *. We converted measurements in previous studies from a mass exponent of 1 to an exponent of 0.67 to correct for allometric relationships between metabolic rate and hatchling mass [79]. Statistical differences were determined using mixed-effects models and Tukey’s pairwise comparisons

During the frenzy, hatchling MMR was always higher than RMR in green (z = 31.65, *p* < 0.001), olive ridley (z = 8.18, *p* < 0.001), and flatback sea turtle hatchlings (z = 6.87, *p* < 0.001) (Fig. 3). This continued to be the case 4-weeks post-frenzy for olive ridley (z = 5.08, *p* < 0.001) and flatback hatchlings (z = 9.76, *p* < 0.001) (Fig. 4). During the frenzy, AMR was higher than RMR in loggerhead (z =  − 3.069, *p* = 0.001) and green sea turtle hatchlings (z =  − 9.84, *p* < 0.001; Fig. 3). The difference between AMR and RMR was maintained in green sea turtles 3-weeks post-frenzy (z = -2.64, *p* = 0.04) and in loggerheads 1-week post-frenzy (z =  − 3.47 *p* = 0.003; Fig. 4).

In leatherbacks, there was no difference between AMR and RMR during the frenzy (z =  − 1.07, *p* = 0.71) or 6-weeks post-frenzy (z = 0.05, *p* = 1). Additionally, in leatherbacks crawling metabolic rate (CMR) did not differ from RMR (z = 1.82, *p* = 0.26) or from AMR (z = 1.18, *p* = 0.96). In loggerheads, CMR was higher than RMR (z = 2.6, *p* = 0.046) but did not differ from AMR (z =  − 1.43, *p* = 0.48; Fig. 3).

CMR in green sea turtle hatchlings was higher than RMR (z = 3.37, *p* = 0.004), but lower than both MMR (z =  − 5.64, *p* < 0.001) and AMR (z =  − 4.99, *p* < 0.001) during the frenzy. However, MMR and AMR did not differ in green turtles during their frenzy (z =  − 0.06, *p* = 0.99; Fig. 4).

### Inter-specific comparisons of metabolic rates

Species differed significantly in their metabolic rates during the frenzy. Leatherback hatchlings had the highest resting metabolic rate (RMR) and olive ridley hatchlings the lowest, while flatback and green hatchlings had intermediate RMR. Loggerhead hatchling RMR did not differ from any of the other species’ RMR (Fig. 1, Table 5).

**Table 5:** Results from Tukey’s pairwise comparisons of the resting metabolic rates of flatback, green, leatherback, loggerhead and olive ridley turtles during the frenzy

	Flatback	Green	Leatherback	Loggerhead
Green	z = 0.94, *p* = 0.88			
Leatherback	z = − 4.42, *p* < 0.001*	z = − 5.33, *p* < 0.001*		
Loggerhead	z = − 0.41, *p* = 0.99	z = − 0.8, *p* = 0.93	z = 2.21, *p* = 0.17	
Olive ridley	z = 4.85, *p* > 0.001*	z = 3.99, *p* < 0.001*	z = 7.24, *p* < 0.001*	z = 2.72, *p* = 0.052

Significant results are marked with *

Flatback and olive ridley RMR did not differ 4-weeks post-frenzy (z = 1.29, *p* = 0.7; Fig. 1).

Green hatchlings had lower crawling metabolic rates (CMR) than leatherback hatchlings (z =  − 3.18, *p* = 0.01), but CMR did not differ between green and loggerhead (z =  − 2.11, *p* = 0.22) or leatherback and loggerhead hatchlings (z = 1.06, *p* = 0.83).

While swimming routinely during the frenzy, the oxygen consumption of green hatchlings was higher than leatherback hatchlings (z = 3.02, *p* = 0.02), but loggerhead metabolic rates did not differ from leatherback (z =  − 1.38, *p* = 0.64) or green hatchlings (z = 1.38, *p* = 0.64; Fig. 2).

One-week post-frenzy, loggerhead hatchling AMR did not differ from leatherback (z = 0.36, *p* = 1) or green hatchlings (z = 2.38, *p* = 0.12). Green and leatherback hatchling AMR did not differ 1-week post-frenzy (z = 2.01, *p* = 0.26). Loggerhead and leatherback hatchling AMR did not differ at 6-weeks post-frenzy (z =  − 2.2, *p* = 0.18) but green hatchling AMR was higher than leatherback hatchlings 3-weeks post-frenzy (z = 3.07, *p* = 0.02; Fig. 2).

When swimming maximally during the frenzy, green turtle hatchlings had higher metabolic rates (MMR) than flatback hatchlings (z =  − 11.81, *p* < 0.001), and both were higher than olive ridley hatchling metabolic rates (green: z = 14.82, *p* < 0.001; flatback: z = 3.54, *p* = 0.004) (Fig. 2). Four weeks post-frenzy swimming, flatback hatchlings had higher maximal metabolic rates (MMR) than olive ridley hatchlings (z = 8.44, *p* < 0.001) (Fig. 2).

Effect sizes among species, behavioural stages and activity levels are shown in Table 6.

**Table 6:** Effect sizes (Hedge’s g) of inter- and intra-specific comparisons of mass-specific metabolic rates

	Flatback	Green	Leatherback	Loggerhead	Olive Ridley
Flatback	**RMR:** FR-4w PF 2.1* **MMR:** FR-4w PF 0.05* **Frenzy:** RMR-MMR 1.22* **4w PF:** RMR-MMR 2.61*	**Frenzy:** RMR *0.24* MMR 2.65*	**Frenzy:** RMR 3.53*	**Frenzy:** RMR *0.22*	**Frenzy:** RMR *2.00* MMR 0.38* **4w PF:** RMR 2.00* MMR 1.56*
Green		**RMR:** FR-3w PF 0.94* **AMR:** FR-1w PF *1.5* FR-3w PF 0.16* 1w PF-3w PF *0.38* **Frenzy:** RMR-CMR 1.94* RMR-AMR 7.54* RMR-MMR 4.39* CMR-AMR 1.23* CMR-MMR 1.92* AMR-MMR *0.52* **3w PF:** RMR-AMR 0.99*	**Frenzy:** RMR 3.31* CMR 0.09* AMR 1.89* **1w PF:** AMR *1.31* **3w PF:** AMR 1.11*	**Frenzy:** RMR *0.04* CMR *0.06* AMR *1.53* **1w PF:** AMR *0.98*	**Frenzy:** RMR 1.44* MMR 3.21*
Leatherback			**RMR:** FR-6w PF *1.7* **AMR:** FR-1w PF *1.17* FR-3w PF 2.5* FR-6w PF 2.26* 1w PF-3w PF *2.19* 1w PF-6w PF *1.58* 3w PF-6w PF *1.03* **Frenzy:** RMR-CMR *0.68* RMR-AMR *0.77* CMR-AMR *0.21* **6w PF:** RMR-AMR *0.06*	**Frenzy:** RMR 2.31 CMR *0.06* AMR *0.6* **1w PF:** AMR *0.03* **6w PF:** AMR *1.79*	**Frenzy:** RMR 6.28*
Loggerhead				**RMR:** FR-1w PF *0.23* **AMR:** FR-1w PF 1.43* FR-4w PF 3.22* FR-6w PF *1.94* 1w PF-4w PF *0.56* 1w PF-6w PF *0.08* 4w PF-6w PF *1.19* **Frenzy:** RMR-CMR 1.77* RMR-AMR 3.94* RMR-MMR *2.09* CMR-AMR *0.86* **1w PF:** RMR-AMR 3.94*	**Frenzy:** RMR *2.16*
Olive Ridley					**RMR:** FR-4w PF *0.37* **MMR:** FR-4w PF 1.07* **Frenzy:** RMR-MMR 2.09* **4w PF:** RMR-MMR 1.84*

Intra-specific comparisons are listed diagonally, and inter-specific comparisons are listed in the cell that corresponds to the two species being compared. We mark comparisons that were statistically different in our linear-mixed effect models with *

### Aerobic scope

**Fig. 5:**
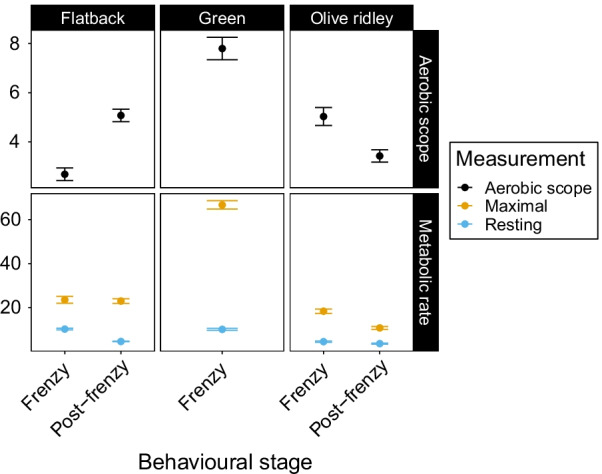
Comparison of mean sea turtle hatchling aerobic scopes during the frenzy and 4-weeks post-frenzy. Error bars represent standard errors. We present aerobic scopes (black) on top and resting (blue) and maximal metabolic rates (yellow) on the bottom. Metabolic rates are reported as μL/g^0.67^/min. Statistical differences between aerobic scopes within species are signified with *. Letters represent statistical similarities among species’ aerobic scopes during the frenzy and post-frenzy, respectively. Statistical differences were determined using mixed-effects models and Tukey’s pairwise comparisons

Our linear mixed effects model detected differences in aerobic scope among species (F_2,383_ = 49.299, *p* < 0.0001), but not among behavioural stages (F_1,383_ = 1.29, *p* = 0.257). However, there was a significant interaction between species and behavioural stage (F_1,383_ = 32.999, *p* < 0.0001), so we used pairwise comparisons to identify significant interactions. During the frenzy, green hatchling aerobic scope was higher than both flatback (t_383_ =  − 11.06, *p* < 0.001) and olive ridley hatchling aerobic scope (t_383_ = 5.81, *p* < 0.001). Flatback hatchling aerobic scope was higher than olive ridley hatchlings (t_383_ =  − 4.79, *p* < 0.001). Post-frenzy, flatback hatchling aerobic scopes were higher than olive ridley hatchling aerobic scopes (t_383_ = 3.337, *p* = 0.003). Flatback aerobic scope was higher post-frenzy than during the frenzy (t_168_ = -5, *p* < 0.0001) but olive ridley aerobic scope was higher during the frenzy (t_177_ = 3.173, *p* = 0.002) (Fig. 5). We did not include leatherback or loggerhead turtles in our analysis of aerobic scope because we did not measure MMR in these two species and thus cannot determine their maximum increase in metabolic rate above resting.

## Discussion

Our objective was to measure and compare the metabolic rates of five different species of sea turtles at different activity levels during their early life in their frenzy and post-frenzy behavioural stages. We used two different respirometry techniques to measure hatchling metabolic rates and found no effect of respirometry technique on metabolic rates. Thus, the use of closed and open respirometry, in our and other studies, both provide reliable measures of metabolic rates. When examining ontogenetic changes in mass-specific metabolic rates, hatchlings that were swimming maximally always consumed more oxygen per minute during the frenzy than post-frenzy, but the change from frenzy to post-frenzy of resting metabolic rate and when swimming routinely varied among species. Throughout this discussion we refer to mass-specific metabolic rates unless stated otherwise.

### Change in oxygen consumption between behavioural stages

#### Resting metabolic rate

Olive ridley, leatherback and loggerhead hatchlings maintained high post-frenzy resting metabolic rates (RMR) that were similar to those during their respective frenzy rates, flatback hatchlings experienced a decrease in RMR after the frenzy, and green hatchlings experienced an increase in RMR after the frenzy. Generally, mass-specific RMR in sea turtles decreases as mass increases [18, 26], but we observed the opposite response in green turtle hatchlings. This increase in RMR post-frenzy may reflect the faster growth rates of green hatchlings in our study compared to other species. Green hatchlings increased in mass by an average of 22% of their initial body mass per week (4.8 g per week) compared to the other species in our study that increased in mass by an average of 4–17% of their initial body mass per week. The intraspecific variation in green hatchling post-frenzy RMR was relatively high and may result from factors including maternal or genetic effects that we didn’t measure here [27]. In contrast, flatback hatchlings experienced a decrease in RMR post-frenzy. Flatback hatchling are entirely neritic [28] and they are thought to forage in murky, turbid waters [29]. Thus, the reduction in flatback RMR may reflect their patchily distributed or hard to find prey placing greater constraints on their energy consumption, compared to other species that associate with sargassum mats and may have more predictable and consistent access to food [30].

In previous studies [18, 19], leatherback hatchlings have also shown reductions in metabolic rate during routine swimming (AMR), maximal swimming (MMR) and RMR post-frenzy compared with the frenzy. Leatherback turtles are entirely pelagic from the time hatchlings leave their natal beaches; they swim continuously during foraging [31–33] and are not thought to associate with oceanic gyres like other species [34]. Thus, the reduction in metabolic rate observed in leatherbacks in other studies potentially allows greater conservation of energy when foraging for patchy prey, similar to flatback hatchlings [35–37]. The statistically similar RMR of leatherback hatchlings during the frenzy and post-frenzy in our study may be the result of low sample sizes and high intraspecific variation. Alternatively, the similar RMR of leatherback hatchlings during the frenzy and post-frenzy may result from our post-frenzy measurements occurring 6 weeks post-frenzy compared to 1–4 weeks post-frenzy in previous studies [18, 19]. Leatherbacks are thought to be faster growing than other sea turtle species, possible because of the high assimilation efficiency of their gelatinous prey [38]. Thus, the initial drop in RMR 1- to 4-weeks post-frenzy and subsequent increase back to levels similar to the frenzy at 6-weeks post-frenzy may represent the transition of leatherback hatchlings from relying mainly on yolk reserves to feeding on gelatinous prey. Indeed, leatherback hatchlings retain approximately 6% of their yolk reserves 4–6 weeks post-emergence [18]. Both loggerhead and olive ridley hatchlings had similar RMR during the frenzy and post-frenzy periods. Olive ridley and loggerhead turtles are the most closely related species in our study and thus, the similarities in the ontogeny of their RMR may represent phylogenetic inertia. Both species inhabit oceanic waters as post-hatchlings before inhabiting neritic waters as larger juveniles and adults, as do green turtles [39], suggesting that these three species may maintain high RMR to facilitate faster growth rates and larger body sizes before migrating to neritic waters as juveniles and subadults.

#### Metabolic rate during routine and maximal swimming

When hatchlings were encouraged to swim maximally by simulating a predation event, both olive ridley and flatback hatchlings decreased in MMR from frenzy to post-frenzy. Similarly, when hatchlings swam of their own volition green, leatherback and loggerhead hatchlings exhibited a general decrease in AMR. This reflects the hatchings’ transitions from the frenzy, during which hatchlings attempt to escape predator-dense waters, to the post-frenzy, when hatchlings can reduce their activity levels in deeper, less predator-dense pelagic waters [40]. However, flatback hatchlings still experience a small decrease in MMR post-frenzy, despite not entering pelagic waters and remaining in neritic waters post-frenzy [39]. Flatback hatchlings in our study were tested while swimming maximally at the surface but when tracked in situ, flatback hatchlings were capable of swimming faster than green sea turtles when motivated [29] and they may utilise anaerobic pathways at greater rates than other sea turtle species [14]. Additionally, flatback hatchlings generally perform slow dives when feeding, potentially to more effectively detect and maintain contact with food patches in murky, turbid waters [29]. They are capable of making repeated dives, only spending short periods at the surface to replenish their oxygen supply [29]. Therefore, maintaining high MMR, similar to levels during the frenzy, may help facilitate the rapid replenishment of oxygen in foraging flatback hatchlings and quick removal of by-products from anaerobic energy pathways. Once underwater, sea turtles exhibit a number of dive responses, including reduced heart rates, that decrease their oxygen usage and maximise the time that they can remain submerged [41]. Thus, high MMR allow flatback hatchlings to quickly replenish their oxygen stores, minimising their time at the surface, before performing slow, energy efficient dives that allow them to maintain contact with prey in murky, turbid waters.

In contrast to our study, Jones et al. [18] found that olive ridley hatchlings had higher MMR post-frenzy than during the frenzy. The different geographic locations from which the eggs were collected may have contributed to the differences between our findings: hatchlings of the population of olive ridley turtles in the Tiwi Islands from which we collected eggs disperse into the relatively shallow Timor and Arafura seas [42], compared to the eastern Pacific ocean off the coast of Costa Rica, where the olive ridley hatchlings in the study by Jones et al. [18] disperse. Tiwi Island olive ridleys are likely to experience higher predation rates during dispersal than hatchlings from Costa Rica because shallow waters generally lead to increased predation rates [40]. Thus, Tiwi Island olive ridley turtles may have undergone selection for higher frenzy MMR to fuel their extended dispersal through shallow waters compared to eastern Pacific olive ridleys. An alternative cause of observed differences in MMR between our study and that of Jones et al. [18] is that 4-week-old olive ridley hatchlings in our study increased in mass by approximately 2 g, compared to the 6 g increase observed by Jones et al. [18]. Two possible explanations are that they were underfed, although this is unlikely because our hatchlings were fed ad libitum, or their rate of feeding was suppressed in captivity. Another is that olive ridley hatchlings in our study did not feed until approximately 12 days post-emergence, compared to the usual 5–7 days [43, 44]. The delayed commencement of feeding in Tiwi Island turtles may have resulted in reduced growth rates, despite Tiwi Island hatchlings initially being heavier (16.46 ± 0.44 g) than Costa Rican hatchlings at emergence (13.2 ± 0.08 g [18]). Thus, the ontogenetic differences in MMR between these two populations may not only reflect genetic, ecological and evolutionary differences but also differences in hatchling quality.

### Comparisons of metabolic rates at different activity levels

AMR, CMR and RMR did not always significantly differ. While the difference between AMR and RMR and also CMR with RMR in green and loggerhead hatchlings likely reflects the near maximal swimming and crawling effort of dispersing sea turtle hatchlings, leatherback AMR, CMR and RMR were similar during both the frenzy and the post-frenzy. Leatherback hatchlings have a relatively low cost of swimming [18] due to their slow, continuous-swimming behaviours. They also grow quickly compared to other sea turtle species [38, 45] and the extra energy demands of faster growth may potentially explain higher RMR in leatherbacks. Thus, elevated RMR and low AMR led to leatherback hatchlings exhibiting little difference in oxygen consumption at rest and during routine swimming. As AMR was measured when hatchlings were swimming without being motivated to swim by mimicking a predation event, it is likely that we measured the AMR of leatherbacks at a range of different intensities. Differences in ‘motivation’ to swim may therefore have resulted in large standard deviations in our data and resulted in no statistical difference between RMR and AMR in leatherback hatchlings.

In different species, the relationship between CMR and AMR or MMR differed slightly to that of AMR and RMR. Loggerhead and leatherback hatchling CMR did not differ from AMR but in green hatchlings, CMR was lower than AMR and MMR. Aerobic metabolism has been shown to be an important energy pathway for digging and crawling hatchlings [46–48], but sea turtle hatchlings have also been shown to extensively utilise anaerobic energy pathways during the initial stages of the frenzy, including crawling from the nest to the ocean [14, 49, 50]. Thus, relationships between aerobic and anaerobic energy pathways during crawling, swimming and when at rest have varied among studies. For example, Pereira et al. [14] found that plasma lactate concentrations were highest during crawling and then decreased within the first 2 h of swimming, while Hamann et al. [46] found that plasma lactate peaked once hatchlings had been swimming for 2 h. When measuring both oxygen consumption and plasma lactate, Pankaew and Milton [47] found that oxygen consumption in green and loggerhead hatchlings was higher in crawling hatchlings than those at rest, while oxygen consumption in swimming hatchlings (AMR) was intermediate and did not differ statistically from RMR or CMR. However, plasma lactate concentrations did not differ among resting, crawling or swimming hatchlings. In our study, neither green nor loggerhead hatchlings consumed more oxygen during crawling compared to when swimming routinely.

Potentially, the ‘motivation’ to crawl or swim among individual hatchlings, clutches and species may vary considerably more than previously thought, resulting in large variation in metabolic measurements and overlap among activity levels. Hatchlings may utilise anaerobic pathways during bursts of crawling and digging, and then utilise aerobic pathways when removing accumulated lactate during rest periods [46, 47]. Differences in the intensity of activity periods or the duration of rest periods could alter oxygen consumption, lactate accumulation and oxygen debt. Thus, similarities among activity levels within studies and differences among studies may be the result of differing levels of ‘motivation’ among hatchlings. Further studies that measure both aerobic and anaerobic metabolism simultaneously are needed to further elucidate how the type and duration of different activities influence whether dispersing hatchlings utilise aerobic or anaerobic metabolic pathways, how the utilisation of either pathway influences performance, and how the accumulation of anaerobic by-products influences oxygen consumption and performance. The strength of cues for the hatchlings may influence hatchling crawling and swimming motivation, while sand characteristics may influence how difficult it is for hatchlings to crawl. Genetic, maternal and incubation effects may also influence the relative use of aerobic and anaerobic pathways of hatchlings during dispersal. Hatchling size and terrestrial gait are unlikely to explain the different relationships between CMR and AMR among species, because green hatchlings are intermediate in size compared to loggerhead and leatherback hatchlings, and green hatchlings utilise the same gait i.e., alternating limb crawling [51], as loggerhead hatchlings when crawling [19]. Lastly, green hatchlings complete fewer powerstrokes in each powerstroking bout and have shorter duration powerstroking bouts than either loggerhead or leatherback hatchlings [10, 19]. Thus, higher swimming intensity in green hatchlings is unlikely to be the reason why green hatchling AMR is higher than their CMR, while other species exhibit similar AMR and CMR.

### Comparisons of metabolic rates among species

#### Resting metabolic rate

Olive ridley hatchling RMR was consistently lower than that of other species. Olive ridley hatchlings in our study increased in mass by ~ 2 g compared to ~ 6 g by olive ridleys in Jones et al. [18]. Thus, the lower metabolic rate at rest in olive ridley hatchlings in our study may be linked to the slower growth rates of these hatchlings, although the relationship between RMR and growth rate is currently unclear [27]. The slower growth rate (4% of their initial body mass per week on average) and therefore, the lower RMR of our olive ridley hatchlings may also result from differences among populations (e.g., genetic, maternal, life history) or may be a response to other unmeasured variables. Likewise, leatherback hatchlings generally had higher RMR than other species during the frenzy and post-frenzy, potentially reflecting their faster growth rates [38, 45].

#### Metabolic rate during routine and maximal swimming

Species varied in their oxygen consumption during routine and maximal swimming. However, green sea turtle hatchlings generally had higher AMR and MMR during the frenzy and post-frenzy compared with other species. These results suggest that green sea turtles expend a greater amount of energy during dispersal compared to other sea turtle species [15, 22]. Interestingly, loggerhead AMR was comparable to that of green hatchlings during the frenzy and post-frenzy, although based on effect size, the difference between green and loggerhead hatchling AMR was always quite large (frenzy:1.53, 1-week post-frenzy: 0.98). The green and loggerhead hatchlings that we collected and tested in the USA both emerge from Floridian beaches, and are likely to follow similar dispersal paths along the east coast of the mainland USA, potentially explaining their similar AMR [52–54]. It is unlikely that the size of energy reserves influence metabolic rates, because loggerhead hatchlings have been shown to have larger residual yolk reserves than green hatchlings [55]. In comparison to green and loggerhead hatchlings, leatherback hatchlings exhibited lower AMR compared to other species. Thus, leatherback hatchlings potentially prioritise the duration of time that they can maintain their swimming effort at the expense of the intensity of their swimming effort [8]. Leatherback turtles possess larger flippers relative to their body size than other turtle species and their longitudinal ridges have enhance hydrodynamic performance during swimming, potentially allowing leatherback turtles to swim more energy-efficiently than other sea turtle species [56].

### Variation in aerobic scope among species and behavioural stages

We were able to measure both RMR and MMR of flatback, green and olive ridley hatchlings. These two measurements represent the aerobic scope, or the capacity of hatchlings to elevate their metabolic rate above maintenance levels [57, 58]. Thus, changes in these two measures reflect the physiological limits for hatchlings in terms of their minimum and maximum energy expenditure, although interpretations of aerobic scope should be taken with some caution. Aerobic scopes have generally been shown to increase as body mass increases, both within [59] and among species [60, 61]. However, our study did not observe a consistent increase in aerobic scope with body mass among species. Potentially, this may be the result of ontogenetic changes in our hatchlings resulting in inconsistent changes in aerobic scope, as seen in teleosts [59]. Thus, we would expect aerobic scopes to increase as our hatchlings continue to grow [19, 62]. In comparison to our study, Jones et al. [18] observed an increase in olive ridley aerobic scope over the same life stages as our study. It is possible that factors such as hatchling quality, housing or incubation conditions or population differences may be responsible for this difference. Some authors have suggested that sedentary animals are likely to have higher aerobic scopes because they have lower RMR resulting from inactivity and higher MMR because of a greater ability to exert periods of maximal activity than constantly active individuals [63]. Conversely, Jackson and Prange [62] and Weibel et al. [61] proposed that animals with an increased ability to migrate will have higher aerobic scopes because of a greater capacity to increase their energy consumption. However, there is no clear connection between aerobic scopes and migration length or the propensity to migrate [18, 64]. Thus, the ecological relevance of aerobic scopes may depend on each species’ behaviours [65] and remains uncertain overall. In our study, green sea turtles had the highest aerobic scopes during the frenzy, largely because of their extremely high MMR (Fig. 5). Although flatback hatchlings had higher MMR and RMR than olive ridleys, their aerobic scope was lower than that of olive ridleys. Flatback hatchling mean swim thrust decreases rapidly during the first 24 h of the frenzy compared to green hatchlings [15, 22], supporting the theory of Jackson and Prange [62] that reduced aerobic scopes may reflect a decreased need to migrate. Thus, it appears that flatback hatchlings may not expend as much energy during dispersal as green or olive ridley hatchlings and that their low aerobic scopes during the frenzy are representative of their shortened migration into neritic waters [39] compared to pelagic species that undergo longer migrations and have greater aerobic scopes. Flatback hatchling aerobic scope increases post-frenzy, potentially reflecting their increased need to rapidly replenish oxygen stores during short breathing intervals when foraging for food in murky, turbid waters [29].

### Comparing hatchling metabolic rates among studies

Oxygen consumption rates in our study were consistently higher than those measured by Prange and Ackerman [66], Davenport and Oxford [67] and Lutcavage and Lutz [68]. These differences may have resulted from the methodology and equipment available in those studies, or from differences in genetics, incubation conditions, acclimation conditions, and housing conditions. Lutcavage and Lutz [68] housed their leatherback hatchlings at 20 °C and acclimated hatchlings at 24 °C before respirometry testing, compared to the warmer housing temperatures in our study (24–28 °C), probably contributing to the higher metabolic rates we measured. Metabolic rates in Clusella Trullas et al. [69] measured at ~ 27 °C, were consistently higher than those in our study, likely because Clusella Trullas et al. [69] used doubly-labelled water to measure metabolic rates, which may not be a feasible method of determining differences among activity levels, because doubly-labelled water estimates energy consumption over a time period, that may be composed of multiple activities [70]. Differences in incubation conditions may also explain variation in metabolic rates between our study and others. Most studies on hatchling metabolic rates do not report incubation conditions, despite incubation conditions having been shown to influence metabolic rates in hatchling turtles [71]. In our study, mean incubation temperatures of olive ridley (29.3 °C), flatback (29.43 °C) and Malaysian green hatchlings (29.45 °C) were all within 0.15 °C of each other. Natural nest temperatures for green, leatherback and loggerhead hatchlings in Florida were not recorded. Additionally, differences in the time hatchlings were given between pipping the egg and being tested could alter frenzy metabolic rates.

The metabolic rates of hatchlings from Jones et al. [18] were consistently lower than hatchlings in our study during the frenzy (Fig. 3). Hatchlings in Jones et al. [18] emerged from natural nests and were allowed to crawl to the ocean before being collected by hand and then tested. Studies that incubate eggs in the laboratory often allow hatchlings to rest in the incubator for 24–48 h to imitate natural behaviour and yolk utilization. Hatchlings that emerge from the nest and spend time crawling could differ in their oxygen consumption compared to hatchlings that do not undertake these activities. The post-frenzy metabolic rates in our study were not consistently higher or lower than those in Jones et al. (2007) (Fig. 4), suggesting that differences among studies are unlikely to be the result of differences in methodology, and may instead reflect variation among populations as shown by differences in olive ridley growth rates. Lastly, metabolic rates in [19] were consistently higher than those in our study, although they were closer in value during the frenzy than during the post-frenzy when hatchling metabolic rates in our studies were closer to those in Jones et al. [18]. However, the metabolic rates in our study were generally similar to metabolic rates measured in other studies (Figs. 3 and 4), with differences among studies likely reflecting the differences mentioned above. Thus, the metabolic rates measured in our study fall within a similar range to other studies, suggesting that the metabolic rates in our study provide a strong indicator of the energetic demands facing hatchlings during the frenzy and post-frenzy periods. Differences between our study and other studies likely reflect differences among populations, species, methodology and housing and incubation conditions. There has only been one in-depth review of sea turtle metabolic rates [26], but future reviews, preferably meta-analyses, should consider the potential sources of variation among species, populations and studies listed above.

### Conclusion

The mass-specific metabolic rates that we measured varied by behavioural stage, activity level, and species. These differences are largely consistent with ecological and life history differences among species. Leatherback hatchlings exhibited similar metabolic rates during rest and routine swimming, and reduced their metabolic rates as they transitioned from the frenzy to the post-frenzy, possibly reflecting their efficient and continuous swimming behaviours. In contrast, flatback hatchlings exhibited only a small decrease in maximal metabolic rates from the frenzy to the post-frenzy. With their completely neritic life history, high MMR may facilitate the quick replenishment of oxygen when foraging in murky waters. Green, loggerhead and olive ridley hatchlings all experienced a drop in metabolic rate during routine or maximal swimming post-frenzy, likely reflecting the transition from dispersal to foraging behaviours. However, changes in RMR varied among the three species, potentially reflecting differences in post-frenzy growth rates. We report comparisons among five of the seven extant species, characterize their early-life metabolic rates and provide the foundations for links between the physiology and ecology of sea turtles. The ecological significance of each species’ metabolic rates will be become clearer as the dispersal paths and distances of different species and populations are determined, but the metabolic rates measured here provide insight into potential differences in dispersal length among species. Our study provides further insight into the ecological significance of aerobic scopes, suggesting that reduced aerobic scopes limit the ability of species to migrate.

## Methods

### Types of respirometry used

In this study we used two methods for measuring oxygen consumption: closed and open flow respirometry (Table 7). Closed respirometry requires creating a chamber with a constant volume and circulating air from the chamber containing the animal through the oxygen analyser and back into the chamber. As oxygen cannot enter this closed system, it is possible to record the fall in oxygen partial pressure within the chamber as it is consumed by the animal. Open flow respirometry draws air continuously from an external source (generally the atmosphere or from a tank), through the chamber containing the animal, then through the oxygen analyser before expelling the air back into the atmosphere. By comparing the concentration of oxygen in the air entering and exiting the chamber, it is possible to calculate the oxygen consumption of that animal. Open flow systems allow measuring metabolic rates over longer periods because there is a continual flow of oxygen into the chamber throughout testing and so local depletion of oxygen does not occur.

**Table 7:** Summary of the methodology used to test each species’ oxygen consumption

	Population	Egg incubation	Closed respirometry (2017/18)		Closed respirometry (2010)	Open flow respirometry (1996, 1999, 2000 & 2001)		
			RMR	MMR	AMR	RMR	CMR	AMR
Respirometer volume/dimensions			375 mL	1000 mL	35 × 35 cm (plexiglass) or 50.8 × 25.4 (glass)	470 mL	25 × 20 cm (glass). Filled with seawater for AMR testing & empty for CMR testing	
Test duration			20 min	15 min	Green: 20 ± 4 min Leatherback: 55 ± 7 min Loggerhead: 27 ± 4 min	90 min	40 min	90 min
Oxygen analyser			PASCO PS-2126A		Applied Electrochemistry O2 Analyser S-3A			
Flatback (*Natator depressus*)	Australia	Incubators- 29.43 ± 0.11ºC	Frenzy & Post-frenzy	Frenzy & Post-frenzy				
Green (*Chelonia mydas*)	Malaysia	Hatchery- 29.45 ± 0.54 ºC	Frenzy	Frenzy				
	USA	*In situ*- temperature not recorded			Frenzy	Frenzy & Post-frenzy	Frenzy	Frenzy & Post-frenzy
Olive Ridley (*Lepidochelys olivacea*)	Australia	Incubators- 29.30 ± 0.03 ºC	Frenzy & Post-frenzy	Frenzy & Post-frenzy				
Leatherback (*Dermochelys coriacea*)	USA	*In situ*- temperature not recorded			Post-frenzy	Frenzy & Post-frenzy	Frenzy	Frenzy & Post-frenzy
Loggerhead (*Caretta caretta*)	USA	*In situ*- temperature not recorded			Post-frenzy	Frenzy & Post-frenzy		Frenzy & Post-frenzy

We list the activity level and behavioural stage that was measured for each species and technique

### Metabolic rates measured

We measured metabolic rate in turtles that were resting (RMR), crawling (CMR) and swimming, both routinely (AMR) and maximally (MMR). Turtles were defined as resting when stationary (only breathing) within the respirometry chamber. Turtles were defined as crawling when actively moving around an empty, dry respirometry chamber. Turtles were considered to be swimming either routinely or maximally: routine swimming (AMR) was assigned when turtles swam without encouragement or prodding, and maximal swimming (MMR) was assigned when turtles were tapped on the carapace with a piece of padded wire to mimic a predation event under natural conditions (Jones et al., 2007). In addition, we measured metabolic rates of hatchlings during the frenzy period, defined by two criteria. First, to be classified as being in the frenzy period hatchlings must have been tested either within 24 h of emerging from natural nests or within 72 h of hatching from the egg when incubated in the laboratory. Sea turtle hatchlings generally take 3–7 days to emerge from the nest and enter the frenzy [48, 72]. Thus, we allowed hatchlings incubated in the laboratory 48–72 h to internalise their yolk and begin the frenzy. Second, to be considered in the frenzy period, RMR and CMR must have been measured in hatchlings that were naïve to the water. For AMR and MMR, measurements must have been made within the first 2 h of hatchlings being introduced to water, as this is when hatchlings exhibit the highest oxygen consumption rates [7].

### Egg collection and incubation

We collected and measured the oxygen consumption of five sea turtle species from four locations using two different respirometry techniques. Thus, it is necessary to describe how hatchlings were collected and tested for each combination of species, location and technique. Among- [18, 49] and within-species [27, 73] comparisons are commonly conducted in multiple hatchling traits, but comparisons of different techniques are less common. Measuring metabolic rates of sea turtle species from several locations using multiple respirometry techniques, like in our study, allows us to evaluate the equivalence of each technique, and results in greater confidence in the reliability of our metabolic rate measurements.

#### Closed respirometry- flatback, green & olive ridley turtle hatchlings

We collected olive ridley and flatback sea turtle eggs in Australia from the Tiwi Islands, NT in 2017 and Curtis Island, QLD in 2018, respectively. We patrolled nesting beaches at night looking for nesting females and collected the eggs as they were laid or just after oviposition if we found the female covering the nest. We collected 30 eggs from each of six females per species. The eggs were vacuum-sealed in bags following the protocol of Williamson et al. [74] to maintain embryonic arrest. Eggs were vacuum-sealed within 1 h of oviposition and were sealed for a total duration of 24–72 h. The sealed bags were placed in a cooler lined with vermiculite or bubble wrap and containing ice packs. We then transported the eggs to Monash University, Melbourne, VIC where they were placed into incubators (1602-N Hova-Bator, GQF manufacturing, USA) within a maximum of 72 h since collection. Keeping eggs in an oxygen-free environment during transport prevents the embryonic development that would commence after 12 to 16 h [75] and result in a risk of movement-induced mortality in transit [76].

In incubators eggs were three-quarters buried in sand and incubated at 29.43 ± 0.11 °C and 29.30 ± 0.03 °C) for flatback and olive ridleys, respectively. Moisture concentrations ranged from 4 to 8% moisture w/w. Incubator temperature was monitored daily using fast response temperature probes (PASCO PS-2135, Pasco, USA) buried next to the eggs and we maintained moisture gravimetrically by drying samples of sand and adding evaporated water with a spray bottle. We removed eggs that turned yellow or showed signs of fungus or mould to avoid contamination of other eggs. Once all eggs had formed white spots, we fully covered the eggs with sand.

Green sea turtle eggs were collected from Kijal beach, Malaysia, (4° 20′ 59.99" N, 103° 28′ 59.99" E), approximately 42 km from the Lang Tengah Turtle Watch hatchery in June 2018. The eggs were transported to the shaded hatchery in buckets lined with sand and buried in the centre of a 1 m^2^ plot with the bottom of the nest at a depth of 70 cm. We collected entire clutches from 20 nesting females and all nests were reburied within six hours of oviposition. We recorded nest temperature (29.45 ± 0.54 °C) with Thermocron ibuttons (DS1921-G#F50, Temp-log Australia, accuracy ± 1 °C, resolution 0.5 °C) every 3 h throughout incubation. We measured moisture with a probe (PASCO ECH_2_O EC-5, Pasco, USA) and each clutch was maintained at between 4 and 8% moisture (v/v) by adding water with a watering can at the surface. The amount of water required each day was determined during a pilot study in which we watered empty plots with various volumes of water and monitored changes in sand moisture concentration at nest depth.

After emerging from the eggs, olive ridley and flatback hatchlings were allowed 48–72 h to internalise their yolk sac before being measured using electronic scales (± 0.001 g) and being marked on the carapace with unique patterns using non-toxic nail polish. Sea turtle hatchlings generally take 3–7 days to emerge from the nest and enter the frenzy [48, 72], so holding them for two or three days after hatching in an incubator more closely represents the natural situation than testing them immediately after hatching. Once flatback and olive ridley hatchlings had internalised their yolk, we measured the oxygen consumption of hatchlings that exhibited high activity levels such as continuous crawling. Individuals selected for metabolic testing were randomly selected from the high activity hatchlings. Green turtle hatchlings were collected as they emerged from their hatchery nests, selected at random and tested immediately. The same olive ridley and flatback hatchlings were measured for both RMR and MMR.

#### Closed respirometry- leatherback, loggerhead and green turtle hatchlings

Hatchlings were collected as they emerged from natural nests laid in Boca Raton, Florida, USA in June, July and August of 2010. Hatchlings were transported to Florida Atlantic University located 8 km (< 15 min) away, selected at random for testing and weighed using an electronic balance (± 0.01 g) or a Pesola™ scale (± 0.3%).

#### Open flow respirometry- leatherback, loggerhead and green turtle hatchlings

Green, loggerhead and leatherback hatchlings were collected from natural nests laid in Boca Raton, Florida, USA in June, July and August of 1996, 1999, 2000 and 2001. Additional leatherback hatchlings were collected from natural nests laid in Hillsboro Beach, Juno Beach and Jupiter Beach, Florida, USA. Hatchlings were transported to Florida Atlantic University, selected at random for testing and weighed using an electronic balance (± 0.01 g) or a Pesola™ scale (± 0.3%). Florida Atlantic University is located 8 km (< 15 min) from Boca Raton, 10 km (~ 15 min) from Hillsboro Beach and 65 km (~ 45 min) from Juno and Jupiter Beaches.

### Hatchling housing and release

#### Closed respirometry- flatback, green and olive ridley turtle hatchlings

The hatchlings we tested in their frenzy were naïve to water prior to the measurement of their metabolic rates. After measurement had been completed, olive ridley and flatback hatchlings were housed in 3L and 10L plastic tanks or in glass tanks separated with egg crating (12.5 mm grid, Aquasonic, Australia). Tanks were kept clean by a continuous flow-through filtration system consisting of a drum filter (Faivre 60 series, Faivre, France), fluid sand bed filters (RK2 systems, USA), a protein skimmer (RK10AC, RK2 systems, USA), a UV filter (240 W UV steriliser, Emperor Aquatics, USA) and an ozone steriliser (RK300MG, RK2 systems, USA). Water quality was monitored daily using OxyGuard hand-held monitors (Technolab, Australia). Water temperature was maintained between 26 and 27 °C using a heater (3 kW heater, Shego, Germany) and a chiller (FBT175SSD, Toyesi, Australia). Animals were maintained under a day/night cycle of 12 h and provided with UV lighting (Exo Terra Repti Glo 5.0 25 W). Turtles were fed daily with commercial turtle pellets (4 mm Marine float range, Ridley Aquafeed).

Olive ridley and flatback hatchlings were housed for 28 days before being tested for post-frenzy RMR and MMR. After post-frenzy testing was completed, hatchlings were transported back to the site of egg collection and released. Following respirometry, green hatchlings were released on the beach adjacent to the Lang Tengah Turtle Watch hatchery within 24 h of emerging from the nest.

#### Closed & open flow respirometry- leatherback, loggerhead and green turtle hatchlings

Hatchlings in their frenzy were naïve to the water prior to the measurement of metabolic rate. They were held in Styrofoam™ boxes with nest sand and placed in a quiet, dark room prior to testing. After frenzy testing, hatchlings were housed at Florida Atlantic University in clutch-specific tanks with separate water and filter systems for each clutch. Leatherbacks were housed using a tether system that prevented hatchlings from touching the side of the tanks while still allowing swimming in any direction, following the protocol of Jones et al. [77]. Green and loggerhead hatchlings were individually housed in plastic baskets suspended within the larger clutch-specific holding tank. The baskets allowed seawater to circulate via small holes in the side of the baskets but kept hatchlings physically separated. Tank water was approximately the same temperature as the ocean water (range 24 °C–28 °C). Hatchlings were fed daily after day 3 (loggerheads) and day 5 (leatherbacks). Loggerheads were fed a combination of chopped shrimp and an in-house manufactured gel diet [78] and leatherbacks were fed blended squid set in agar gel [38]. Hatchlings were provided with 12 h of full-spectrum radiation daily by UV lighting and were released offshore following testing.

### Measuring metabolic rates

#### Closed respirometry- flatback, green and olive ridley turtles

##### Resting metabolic rate

We tested RMR in air by placing hatchlings in a small, opaque chamber (~ 375 mL) with an O_2_ probe (PASCO PS-2126A, resolution ± 0.025%) recording the change in O_2_ concentration. We used soda lime (Scharlau, Australia) and Drierite™ (Hach, Australia) to remove CO_2_ and H_2_O from the air, respectively. We calibrated the O_2_ probe to the ambient O_2_ concentration (20.9%) before each trial began and checked the system for leaks using N_2_ gas. We began RMR trials once the hatchling became still (determined from no audible sound from the claws or flippers on the glass, generally within 5 min) and we abandoned and then restarted trials if the hatchling became active. Hatchlings remained in the respirometry chamber for 20 min, or if trials were abandoned and restarted, for a total of 30 min including both trials. The O_2_ probe was calibrated before each trial with dry, CO_2_ free air and data was corrected to STP.

##### Maximal metabolic rate

We placed a glass chamber upside-down in seawater, creating a pocket of air between the water and the chamber (~ 1000 mL). We pumped air from the chamber at ~ 200 ml min^−1^ over an O_2_ probe (PASCO PS-2126A, resolution ± 0.025%)) sampling at 2 Hz before returning the air to the chamber. Flow rate was controlled using a variable area flowmeter. The air was scrubbed using soda lime to remove CO_2_ and Drierite™ to remove H_2_O before passing over the O_2_ probe. Hatchlings were placed in elasticised harnesses and tethered to the top of the chamber with fishing line so they could swim but not touch the sides or bottom of the chamber. We placed a light at one end of the chamber to encourage the hatchling to swim unidirectionally. Trials lasted 15 min and to ensure the hatchlings swam maximally, we tapped them gently on the back of the carapace using a bent piece of wire passed underneath the chamber, encouraging a flight response [18]. Before each trial, we ran the system without a hatchling to ensure that background respiration by microorganisms in the water did not alter our measurements. The O_2_ probe was calibrated before each trial with dry, CO_2_ free air and data was corrected to STP.

#### Closed respirometry- leatherback, loggerhead and green turtles

##### Metabolic rate during routine swimming (AMR)

Testing occurred in a 35 cm × 35 cm Plexiglass™ respirometry chamber or a glass and acrylic chamber (loggerheads and leatherbacks) that was 50.8 cm × 25.4 cm. Chambers were filled with seawater so that an air space of 1–2 cm in height was left between the chamber lid and the water. Thus, the air volume during testing could be calculated from the chamber cross-sectional area and the height of the air space. Air from inside the chamber was pumped through an Applied Electrochemistry O_2_ Analyser S-3A (AEI Technologies, Pittsburgh, PN, USA, resolution ± 0.01% O_2_) and recirculated back into the chamber. We replaced the seawater with fresh, autoclaved seawater allowed to come to room temperature between clutches. Thus, we assume that there was no background respiration by microorganisms in the water that might alter our measurements.

Leatherback hatchlings were tested at 20 days, 23 days or 44 days post-emergence. Loggerhead hatchlings were tested at 6 days, 43 days, 51 days or 52 days. Green turtle hatchlings were all tested on the day of emergence. Tank temperature was recorded before each trial. Each hatchling was fitted with a Velcro™ strip attached with Vetbond (3 M, USA), slightly caudal to the longitudinal midpoint along the midline of the carapace. We attached one end of a monofilament line to the Velcro strip and the other to the top of the respirometry chamber. Thus, hatchlings could swim in any direction without touching the walls or bottom of the chamber. Hatchlings were allowed to acclimate for 30 min, while the respirometry system was bypassed and sampled ambient air.

Once the hatchling had acclimated, the system was reconnected and air of known O_2_ and N_2_ partial pressure flowed through a Mass Flow Controller (Sierra Side-Trak 840, Sierra Instruments, USA). Air was scrubbed of water vapor (Drierite™ water absorbent, W.A. Hammond DRIERITE, USA) before being drawn through an Applied Electrochemistry O_2_ Analyser S-3A (AEI Technologies, USA, resolution ± 0.01% O_2_). Data from the mass flow controller and oxygen analyser were recorded at the start and the end of the trial and was analysed using DataCan V Data Acquisition and Analysis Software and Hardware (Sable Systems International, USA). Air was then re-circulated back through the chamber. Respirometer calibration was done using the N_2_ dilution technique (Fedak et al., 1981). VO_2_ data were corrected for analyzer drift and to STP. Leatherback hatchling testing lasted for an average of 55 ± 7 min, green hatchlings for 20 ± 4 min and loggerheads for an average of 27 ± 6 min.

#### Open flow respirometry- leatherback, loggerhead and green turtles

##### Resting metabolic rate

We tested RMR in air by placing hatchlings in an approximately 470 mL black container (~ 10 cm × 7.5 cm) closed with a large rubber stopper fitted with air intake and outflow. Each turtle was allowed to acclimate for 30 min, and hatchling movement was minimised in the small container. Once hatchlings were inactive (determined from no audible sound from the claws or flippers on the glass), we closed the container, and began measuring O_2_ consumption.

Incurrent air was drawn continuously through a hole drilled in the chamber lid into the space between the chamber walls and the water inside the chamber. Air from inside the chamber was drawn through a second hole, passed through a water absorber (Drierite™ water absorbent, W.A. Hammond DRIERITE, Xenia, USA), a Mass Flow Controller (Sierra Side-Trak 840, Sierra Instruments, USA) and an Applied Electrochemistry Oxygen Analyser S-3A (AEI Technologies, USA, resolution ± 0.01% O_2_) before being pumped into the atmosphere. The O_2_ analyser was calibrated before and after each trial with dry, CO_2_ free air (22% N_2_, 78% O_2_ standard) and data was corrected for analyser drift and to STP.

If hatchlings became active, we restarted metabolic measurements. Hatchlings were tested for 90 min. Leatherback, loggerhead and green hatchlings were tested during the frenzy at 0 days of age because hatchlings had naturally emerged from in situ nests. Post-frenzy testing occurred at 45 days of age for leatherbacks, 12 days of age for loggerheads, and 22, 25 or 26 days of age for green turtle hatchlings.

##### Crawling metabolic rate & metabolic rate during routine swimming (AMR)

Testing occurred in a 26 L tank fitted with an acrylic respirometry chamber with the lid sealed with petroleum jelly. For CMR testing, hatchlings were allowed to crawl on a textured glass floor. For AMR testing, hatchlings were allowed to swim of their own volition, without encouragement. The chamber was filled with seawater so that an air pocket of 2 cm in height × 25 cm × 20 cm was left between the chamber lid and the water. Thus, the air volume during testing could be calculated from the chamber cross-sectional area and the height of the air space. Between turtles, we sanitized the tank and replaced the seawater with fresh, autoclaved seawater allowed to come to room temperature. Thus, we assume that there was not background respiration by microorganisms in the water that might alter our measurements.

For AMR testing, each hatchling was fitted with a Velcro strip using Vetbond as described above. Hatchlings were allowed to acclimate for 30 min for CMR & AMR testing. Incurrent air was drawn continuously through a hole drilled in the chamber lid. Air from inside the chamber was drawn through a second hole, passed through a water absorber, mass flow controller and O_2_ analyser as described above for RMR testing. The O_2_ analyser was calibrated before and after each trial with dry, CO_2_ free air (22% N_2_, 78% O_2_ standard) and data was corrected for analyser drift and to STP.

Room temperature was recorded before each trial and hatchlings were tested for 40 min (CMR) or 90 min (AMR). Leatherback, loggerhead and green hatchlings were tested during the frenzy at 0 days of age (CMR & AMR). Post-frenzy (AMR only) testing occurred at 7, 45 or 50 days of age for leatherbacks; 7 or 31 days of age for loggerheads; and 7 or 23 days of age for green hatchlings.

### Data analysis

Hatchlings were tested at ages that ranged from 0 to 52 days of age. Sample sizes in some of these ages were limited, so we combined some age groups to increase statistical power and to be able to make clearer comparisons among different age groups. Hatchlings tested within 72 h of hatching in incubators or within 24 h of emerging from natural nests were designated as frenzy hatchlings. After the frenzy period, hatchlings were allocated to one of four groups determined by the number of days elapsed since the hatchlings entered the frenzy: 1-week post-frenzy (6-, 7- and 12-day old hatchlings), 3-weeks post-frenzy (20-, 22-, 23-, 25-, and 26-days old), 4-weeks post-frenzy (28- and 31-days old) and 6-weeks post-frenzy (43-, 44-, 45-, 50-, 51- and 52-days old).

For closed system respirometry, we calculated oxygen consumption (VO_2_) (µL min^−1^) using the formula:

1 $$VO_{2} = \left( {\left( {\frac{{\% O_{2}^{I} - \% O_{2}^{F} }}{100}} \right)*V} \right)/\left( {t_{F} - t_{I} } \right)$$

where %O_2_^I^ is the initial percentage of oxygen in the respirometer at the start of the trial, %O_2_^F^ is the final percentage of oxygen in the respirometer at the end of the trial, V is the volume of air contained by the respirometer (μL), t_I_ is the time at the start of the trial (min) and t_F_ is the time at the end of the trial (min). When calculating the mass-specific metabolic rates of hatchlings, we used a mass exponent of 0.67 [79] to correct for allometric relationships between metabolic rate and hatchling mass. Allometric relationships between metabolic rates and hatchling mass vary among species and individual mass [79]. The value of 0.67 used in our study was obtained by averaging the mass exponents of multiple sea turtle species measured at similar temperatures to those in our study. Small changes in pressure from carbon dioxide and water vapour removal were compensated for by water level flux. As the bottom seal of the air pocket within the respirometer (for AMR and MMR trials) was formed by the water that the hatchling was swimming in, the water level rose slightly as carbon dioxide and water vapour were removed, resulting in constant pressure.

For open flow respirometry, we calculated oxygen consumption (µL min^−1^) using the formula:

2 $$VO_{2} = \left( {FR*\left( {\frac{{\% O_{2}^{I} - \% O_{2}^{E} }}{100}} \right)} \right)/\left( {1 - \left( {\frac{{\% O_{2}^{I} }}{100}} \right)} \right)$$

where FR is the flow rate (µl/min) of air through the chamber, %O_2_^I^ is the incoming fraction of oxygen in the air entering the chamber and %O_2_^E^ is the fraction of oxygen in the air exiting the chamber. This formula is used to calculate oxygen consumption when H_2_O and CO_2_ are removed and when flow rate is measured before air enters the chamber. Oxygen consumption was calculated every 5 min and then averaged to calculate the mean oxygen consumption over the entire trial. All measurements of oxygen consumption were mass-adjusted, both within- and among-species, for comparisons of metabolic rates among animals of different sizes.

To determine the overall differences in metabolic rate at all activity levels, behavioural stages and species, we used a linear mixed effects model of mass-specific metabolic rate using in the lme4 package in R [80, 81]. We chose mixed effects models to account for our repeated measures of individual hatchlings and for our unbalanced experimental design. Activity (resting, crawling, routine and maximal swimming), behavioural stage (frenzy and post-frenzy) and species (green, leatherback, loggerhead, olive ridley and flatback turtles) were the fixed effects, while test temperature (air for RMR and CMR or water for AMR and MMR) and hatchling ID nested within location and species were the random effects. We included interaction terms for all fixed effects to account for changes in metabolic rate that were dependent on two or more variables (i.e. the change in metabolic rate from frenzy to post-frenzy by species or by activity level).

The data in this study was collected using 3 different respirometry techniques and configurations. Thus, we initially included respirometry technique (open flow, closed (measured in 2010) and closed (measured in 2017–18)) as a fixed effect to account for variation among the different techniques. However, respirometry technique was not a significant variable in our model (F_2,437.1_ = 1.75, *p* = 0.17), so we excluded respirometry technique from our final model.

Our data were not normally distributed, so we ran our linear mixed effects model with a log link function to meet the assumption of normality. All of our fixed effects and interactions were significant, so we explored each fixed effect separately to identify differences between each level of that effect. We constructed pairwise comparisons using Tukey tests in the package ‘emmeans’ to explore each fixed effect separately. We also calculated effect sizes using Hedge’s g to evaluate differences among species, behavioural stages and activity levels.

Aerobic scopes represent the ability of an organism to increase its metabolic rate above resting metabolic rate [18, 62]. True aerobic scopes are determined from maximal and standard metabolic rates (SMR) in ectotherms (basal for endotherms). SMR is defined as the metabolic rate of an ectotherm with no muscular activity and is not actively digesting food, at a specified temperature [82]. However, sea turtle hatchlings utilise yolk reserves for up approximately a week post-hatching. Thus, it is not possible to measure SMR in hatchlings with yolk reserves such as sea turtles. Therefore, we calculated factorial aerobic scopes by dividing MMR by RMR to show ontogenetic differences among species in their ability to increase their metabolic rate above resting levels for dispersal, escaping predation and for chasing prey. Measurements of RMR include the costs of maintenance i.e. SMR, the costs of digestion and the costs of somatic growth.

We examined aerobic scope between behavioural stages using linear mixed effects models to identify differences among species. Behavioural stage and species were the fixed effects and hatchling ID nested within species was the random effect. We constructed pairwise comparisons using Tukey tests in the package ‘emmeans’ to identify how fixed effects differed.

## Data Availability

Data associated with this study can be found at https://doi.org/10.26180/5f50935ad2aab.
